# Phosphatase regulatory subunit MYPT2 knockout partially compensates for the cardiac dysfunction in mice caused by lack of myosin light chain kinase 3

**DOI:** 10.1016/j.jbc.2023.104584

**Published:** 2023-03-07

**Authors:** Tingfei Hu, Hema Kalyanaraman, Renate B. Pilz, Darren E. Casteel

**Affiliations:** Department of Medicine, University of California San Diego, La Jolla, California, USA

**Keywords:** MYPT2, MLCK3, MLC-2v, cardiac function, PP1Cδ

## Abstract

Cardiac contraction is modulated by the phosphorylation state of myosin regulatory light chain 2 (MLC-2v). The level of MLC-2v phosphorylation is dependent on the opposing activities of MLC kinases and phosphatases. The predominant MLC phosphatase found in cardiac myocytes contains Myosin Phosphatase Targeting Subunit 2 (MYPT2). Overexpression of MYPT2 in cardiac myocytes results in a decreased level of MLC phosphorylation, reduced left ventricular contraction, and induction of hypertrophy; however, the effect of knocking out MYPT2 on cardiac function is unknown. We obtained heterozygous mice containing a MYPT2 null allele from the Mutant Mouse Resource Center. These mice were produced in a C57BL/6N background which lack MLCK3, the main regulatory light chain kinase in cardiac myocytes. We found that mice null for MYPT2 were viable and had no obvious phenotypic abnormality when compared to WT mice. Additionally, we determined that WT C57BL/6N mice had a low basal level of MLC-2v phosphorylation, which was significantly increased when MYPT2 was absent. At 12-weeks, MYPT2 KO mice had smaller hearts and showed downregulation of genes involved in cardiac remodeling. Using cardiac echo, we found that 24-week-old male MYPT2 KO mice had decreased heart size with increased fractional shortening compared to their MYPT2 WT littermates. Collectively, these studies highlight the important role that MYPT2 plays in cardiac function *in vivo* and demonstrate that its deletion can partially compensate for the lack of MLCK3.

The sarcomere is the basic contractile unit in myocytes (1). An individual sarcomere is composed of both thin and thick filaments. The thin filament consists of two intertwined chains of actin with regulatory proteins troponin and tropomyosin, whereas the thick filament consists of organized bundles of myosin. One functional unit of myosin contains two myosin heavy chains, two essential myosin light chains (MLCs), and two regulatory MLCs, specifically ventricular regulatory myosin light chain 2 (MLC-2v) (2). The phosphorylation state of MLC-2v alters the binding of the myosin heavy chain head to actin, modulating muscle contraction and relaxation (3, 4). MLC-2v phosphorylation occurs in a gradient, with higher levels in the outer layers at the apex and lower phosphorylation toward the inner layers and the mid-cardiac region (5, 6). This gradient may improve cardiac output by inducing cardiac torsion (5, 6). The importance of MLC-2v phosphorylation is highlighted in mice carrying alanine mutations at the regulatory phosphorylation sites (Ser^14^/Ser^15^); these mice have a shorter lifespan, develop dilated cardiac myopathy, and show decreased cardiac function by 2 months of age (6).

In normal beating hearts, MLC-2v is approximately 40% phosphorylated (7). The phosphorylation of MLC-2v is dependent on the opposing activities of myosin light chain kinases (MLCKs) and myosin light chain phosphatases. The main MLCK in hearts is MLCK3 (aka cMLCK) (8). In mice lacking MLCK3, MLC-2v levels fall to approximately 10% (9), with the remaining phosphorylation likely mediated by MLCK4 or Zip kinase (10, 11). Under basal conditions, MLCK3 KO mice (either germline or induced) develop enlarged hearts and show signs of cardiac dysfunction, including dilated ventricles and decreased fractional shortening (FA) (12, 13). MLCK3 KO mice are also susceptible to stress-induced heart failure (9, 12, 13); however, mice with cardiac-specific MLCK3 overexpression are protected (13).

The predominant cardiac MLC phosphatase is thought to be a heterotrimer, composed of PP1Cδ (catalytic subunit), M21 (a 21 kDa subunit of relatively unknown function), and MYPT2 (a PP1Cδ regulatory and myosin-targeting subunit, which contains binding sites for PP1Cδ, myosin, and M21). MYPT2 was first described as a MYPT1 homolog that was highly expressed in heart (14). Much more is known about MYPT1 function, which is primarily found in smooth muscle cells (3, 7). MYPT1 and MYPT2 both bind the catalytic PP1Cδ subunit and target it to myosin (15, 16). A conserved threonine at the MYPT1/2 C-terminus is phosphorylated by ROCK (or other kinases), and once phosphorylated, residues flanking the phosphorylated residue bind in the PP1Cδ catalytic cleft, inhibiting phosphatase activity (15, 17, 18). Little is known about MYPT2 function *in vivo*. In one study, Mizutani *et al.* (19) found that cardiac myocyte-specific MYPT2 overexpression led to a decrease in phosphorylated MLC-2v and development of hypertrophic cardiomyopathy, with increased end-systolic and end-diastolic volumes and reduced FS. The degree of cardiac dysfunction correlated with the extent of MYPT2 overexpression, demonstrating the importance of balancing the MLCK and myosin light chain phosphatase activities.

In this paper, we characterize MYPT2 KO mice, which were developed by the Mutant Mouse Resource and Research Center. The mice contain a 580-nucleotide deletion in the gene encoding MYPT2 (*Ppp1r12b*), which removes exon 2, creating a frameshift in the mRNA-coding sequence and producing a stop codon 12 amino acids past the splice site. These mice are in a C57BL/6N background, which has been recently shown to lack MLCK3 protein expression, due to a point mutation that produces an out of frame start codon five nucleotides upstream from the normal start codon (20). The C57BL/6N substrains are known to be susceptible to heart failure (21), and the lack of MLCK3 is likely a major contributor to this phenotype. In this study, we found that the knockout of MYPT2 increased MLC-2v phosphorylation levels and improved cardiac function in C57BL/6N mice.

## Results

### Homozygous MYPT2 KO mice are viable

**Figure 1:**
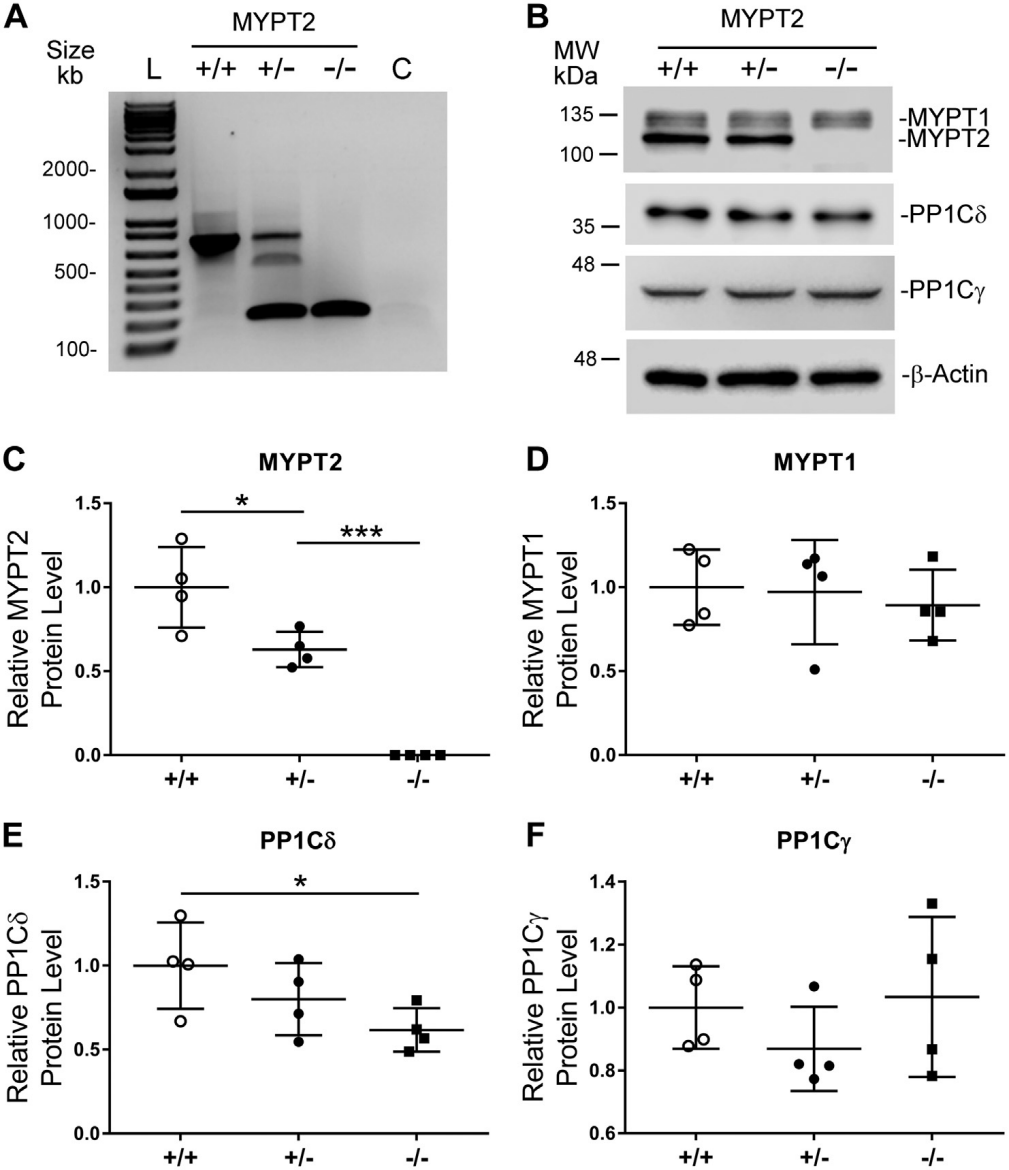
**MYPT2 null C57BL/6N mice are viable and have reduced PP1Cδ in the heart.***A*, agarose gel electrophoresis of MYPT2 genotyping PCR products in WT (+/+), heterozygous (+/−), and homozygous (−/−) mice. The WT allele produces a 775 base pair product, and the mutant allele produces a 195 base pair product. *B*, Western blots of heart lysates from 12-week-old WT (+/+), heterozygous (+/−), and homozygous (−/−) MYPT2 KO mice using pan-MYPT, PP1Cδ, PP1Cγ, and β-actin antibodies. *C–F*, quantification of MYPT2 (panel *C*), MYPT1 (panel *D*), PP1Cδ (panel *E*), and PP1Cγ (panel *F*) expression from Western blots performed as in panel *B*. Graphs represent means ± SD; ∗*p* > 0.05%, ∗∗∗*p* > 0.001 using multiple comparisons (panel *C*) or two-tailed Students *t* test (panel *E*). C, non-template control; L, DNA ladder; MYPT2, Myosin Phosphatase Targeting Subunit 2.

It has been previously shown that global homozygous MYPT1 KO mice are not viable (22), but global homozygous MYPT2 KO mice have not been studied. We bred heterozygous MYPT2 KO mice obtained from the Jackson laboratory and found that homozygous MYPT2 KO mice were viable as determined by genotyping at the time of weaning (Fig. 1*A*). Male and female mice were born at the expected Mendelian ratios, and both male and female MYPT2 KO mice were fertile. MYPT2 KO mice appeared grossly normal up to at least 24-weeks of age and showed no obvious health or behavioral issues when compared to WT mice.

MYPT2 mRNA expression is highest in heart, brain, and skeletal muscle (14). To examine MYPT2 protein expression, we performed Western blots. Since the various MYPT family members share a high degree of sequence similarity, we first assessed the antibody for its ability to detect MYPT1, MYPT2, and MBS85 (another closely related protein). Cell lysates from 293T cells transfected with Flag-tagged expression vectors for MYPT1, MYPT2, and MBS85 were analyzed by Western blotting and probed with an anti-Flag antibody followed by an infra-red IR-Dye 680RD secondary antibody. Imaging was done using a LI-COR Odyssey Fc system. As seen in Fig. S1*A*, anti-Flag blots indicated that the amounts of MYPT1 and MYPT2 proteins were similar, while the amount of MBS85 was approximately 5-fold higher. Probing the same membrane with the anti-MYPT antibody followed by an IR-Dye 800CW secondary, we again observe similar expression levels of MYPT1 and MYPT2 (Fig. S1*B*), but the apparent MBS85 signal was only ∼1.4-fold higher than that of MYPT1/2, indicating that the antibody was less sensitive toward detecting MBS85.

We next probed heart lysates from MYPT2 WT, heterozygous, and KO mice. As expected, MYPT2 expression was lower in heterozygous mice and absent in the homozygous KO mice (Fig. 1, *B* and *C*). The amount of MYPT1 was ∼50% of MYPT2 and was constant in all three genotypes, indicating no apparent compensatory upregulation of MYPT1 (Fig. 1, *B* and *D*). Similar results were seen in lysates from brain and skeletal muscle (Fig. S2, *A* and *B*). Expression of the catalytic PP1Cδ subunit decreased in the MYPT2 KO mice to ∼62% compared to lysates from MYPT2 WT mice (Fig. 1, *B* and *E*). In contrast, PP1Cγ levels did not change (Fig. 1, *B* and *F*).

### MYPT2 KO C57BL/6N mice have increased MLC-2v phosphorylation compared to WT C57BL/6N mice

**Figure 2:**
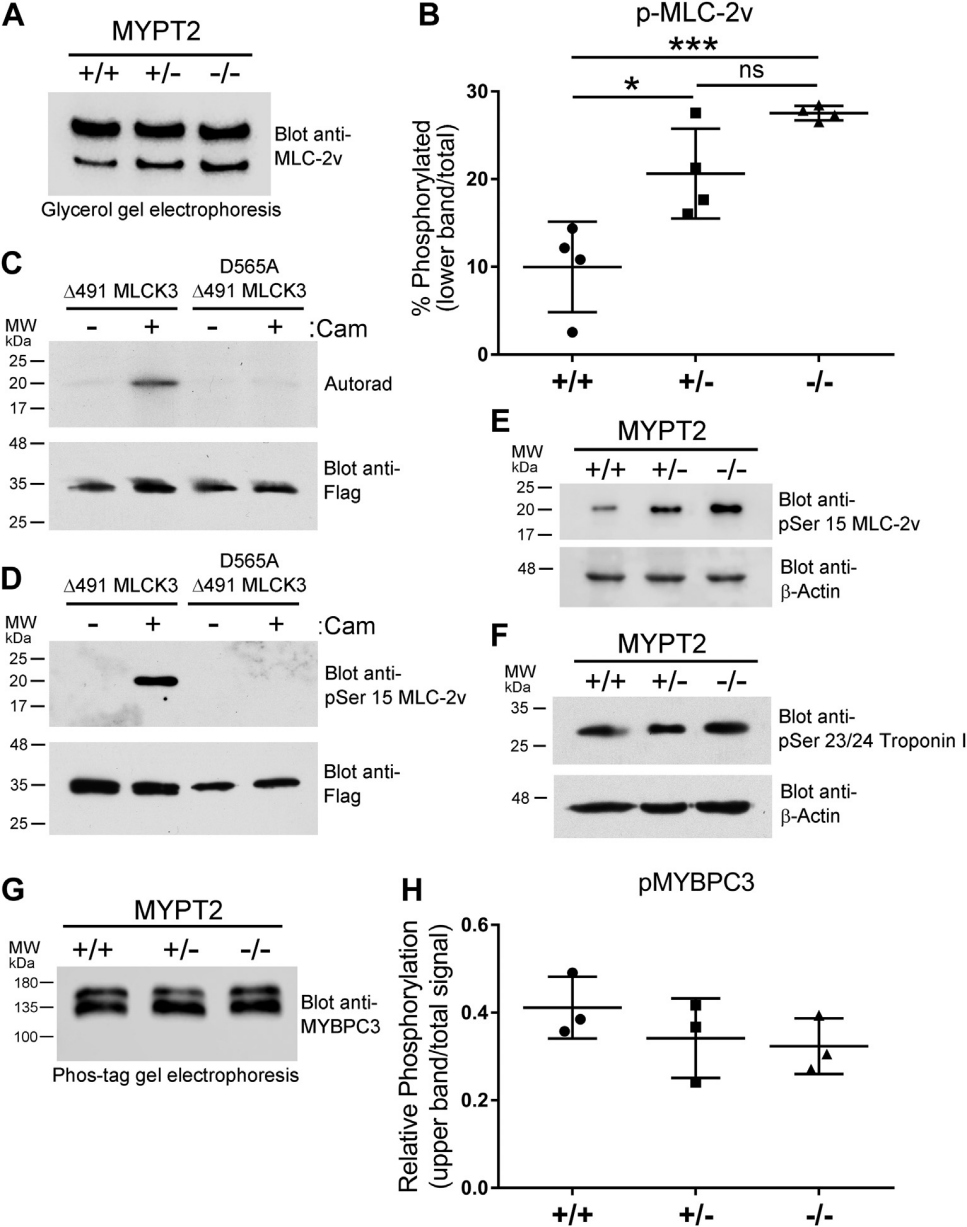
**Phosphorylation of MLC-2v is increased in MYPT2 null C57BL/6N mice.***A*, heart lysates were separated by glycerol/urea gel electrophoresis, in which phosphorylated MLC-2v runs faster than unphosphorylated MLC-2v. Protein were transferred to nitrocellulose membranes and blotted with an anti-MLC-2v antibody. Blots were developed using chemiluminescent substrate and proteins were quantified using a Li-COR Odyssey imaging system. *B*, quantification of four experiments performed as described in panel *A*. *C* and *D*, *in vitro* phosphorylation of purified murine MLC-2v by MLCK3. Phosphorylation was detected by autoradiography (panel *C*) or immunoblotting using an antibody specific for Ser^15^-phosphorylated MLC-2v (panel *D*). Anti-Flag blots show the relative amount of Flag-tagged MLCK3 used in each reaction (*lower blots*). *E* and *F*, heart lysates were separated by SDS-PAGE and analyzed by immunoblotting using the Ser^15^-phosphorylated MLC-2v antibody (panel *E*) or a Ser^23^/Ser^24^-phosphorylated troponin I antibody (panel *F*). *G*, heart lysates were separated by Phos-tag gel electrophoresis and analyzed by immunoblotting using anti-MYBPC3 antibodies. *H*, quantification of three mice per genotype as shown in *G*. Graphs represent means ± SD; ∗*p* > 0.05%, ∗∗∗*p* > 0.001 using multiple comparisons (panel *B*); ns, nonsignificant. MLCK, myosin light chain kinase; MYPT2, Myosin Phosphatase Targeting Subunit 2.

In mice containing both MLCK3 and MYPT2, MLC-2v has a basal phosphorylation level of ∼40% (7), whereas in MLCK3 KO mice, this level is ∼10%, with the residual MLC-2v phosphorylation thought to be mediated by MLCK4 or ZIPK (10, 11). Using glycerol/urea gel electrophoresis and Western blotting, we found that MLC-2v had a basal phosphorylation level of 10% in parental C57BL/6N mice, which is consistent with MLCK3 KO in these mice (Fig. 2, *A* and *B*). We confirmed that our mouse line contained the homozygous MLCK3 mutation which was previously shown to result in a lack of MLCK3 protein expression (Fig. S3*A*) (20). In mice heterozygous for the MYPT2 mutation, MLC-2v phosphorylation increases to 20%, and when MYPT2 is completely knocked out, the level of MLC-2v phosphorylation increases to 28% (Fig. 2, *A* and *B*). Using purified proteins, we found that MLCK3 phosphorylates murine MLC-2v *in vitro*, in a calmodulin-dependent manner: phosphorylation could be detected by autoradiography (Fig. 2*C*) or by immunoblotting using a commercially available phosphospecific antibody raised against human MLC-2v (pSer15) (Fig. 2*D*). No phosphorylation was seen using a kinase-dead D565A-mutant MLCK3 (Fig. 2, *C* and *D*). Human and mouse MLC-2v have different sequences N-terminal to Ser^15^ (Fig. S3*B*), but from our *in vitro* experiments, we can conclude that MLCK3-mediated phosphorylation of mouse MLC-2v can be detected using the antibody raised against human MLC-2v (pSer15). We found a robust increase in MLC-2v phosphorylation in heart lysates from heterozygous and homozygous MYPT2 KO mice when compared to lysates from WT littermates (Fig. 2*E*). Cardiac troponin I is a target of MLCK3 phosphorylation, which in turn modifies the contractile response to MLC-2v phosphorylation (23). We found that the level of troponin I phosphorylation at Ser^23^/Ser^24^ is similar in the presence and absence of MYPT2 in C57BL/6N mice lacking MLCK3 (Fig. 2*F*). Cardiac contraction is also regulated by phosphorylation of myosin-binding protein C3 (MYBPC3) (24). To determine if MYPT2 knockout alters MYBPC3 phosphorylation, we used Phos-tag gel electrophoresis (25). We found that MYBPC3 ran as a doublet, representing different states of phosphorylation, and observed no difference in the overall level of MYBPC3 phosphorylation in the absence or presence of MYPT2 (Fig. 2, *G* and *H*).

### MYPT2 KO C57BL/6N mice have reduced heart weights and lower Myh7 and Tnnt1 mRNA expression than C57BL/6N mice expressing MYPT2

**Figure 3:**
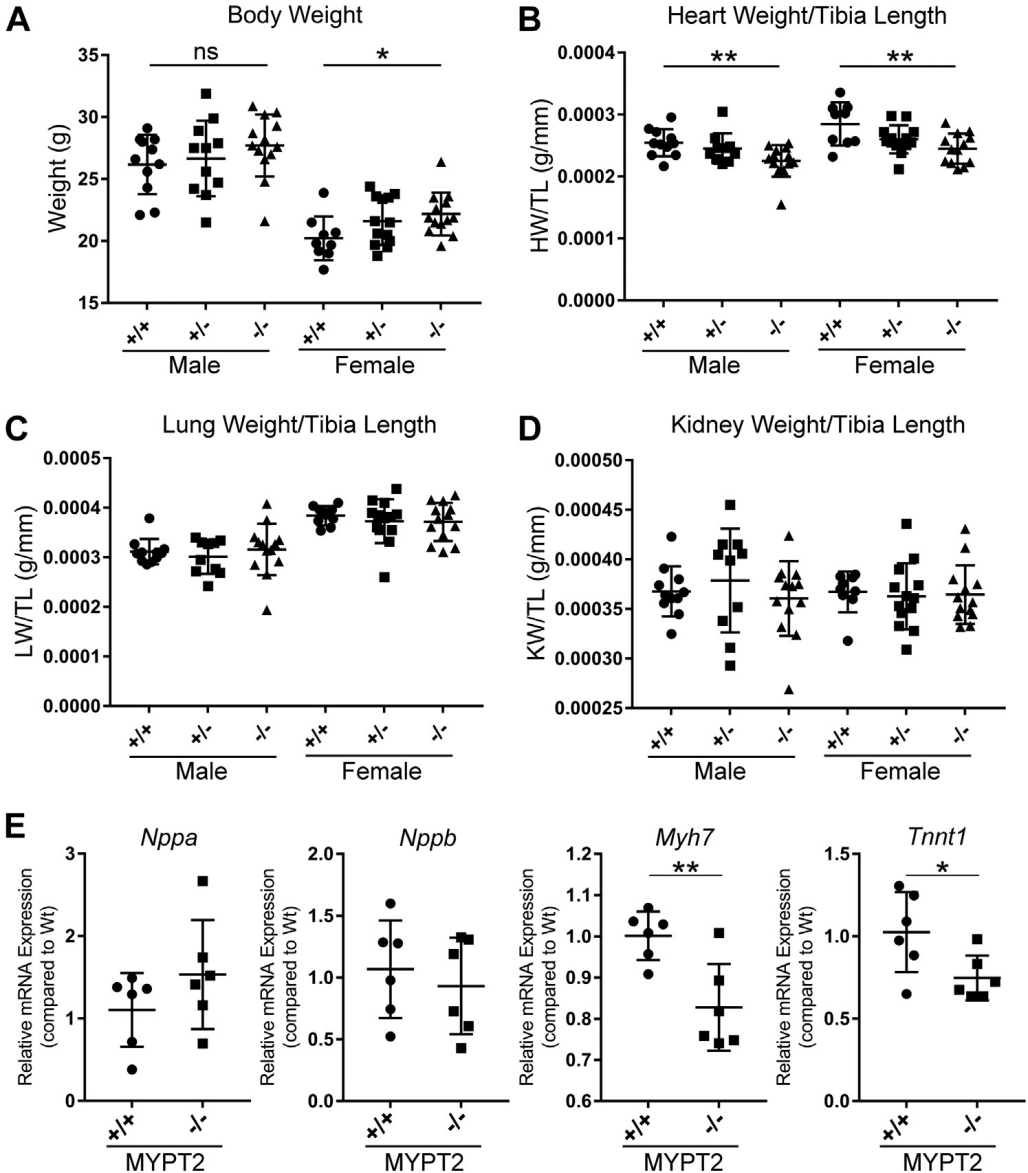
**MYPT2 KO C57BL/6N mice have reduced heart weights.***A–D*, twelve-week-old mice were euthanized using ketamine/xylazine, and organ weights were measured. *E*, quantitative PCRs were performed to measure the expression levels of mRNA for *Nppa*, *Nppb*, *Myh7*, and *Tnnt1* in twelve-week-old mice. Graphs represent means ± SD; ∗*p* > 0.05%, ∗∗*p* > 0.01 using two-tailed Students *t* test; ns, nonsignificant. MYPT2, Myosin Phosphatase Targeting Subunit 2.

MLCK3 KO mice have larger hearts than WT mice (12, 26). We measured the body and organ weights in 12-week-old mice and found that body weight (BW) was increased in female homozygous MYPT2 KO mice, with a similar trend seen in male mice (Fig. 3*A*). The ratio of heart weight to tibia length was significantly decreased in the MYPT2 KO mice (Fig. 3*B*). However, the ratios of lung (Fig. 3*C*) and kidney (Fig. 3*D*) weights to tibia length were similar in all three genotypes.

We used quantitative PCR to measure relative mRNA expression of four key genes involved in cardiac remodeling: atrial natriuretic peptide (*Nppa*), brain natriuretic peptide (*Nppb*), myosin heavy chain polypeptide 7 (*Myh7*), and troponin T1 (*Tnnt1*). We found that *Nppa* and *Nppb* transcript levels did not significantly differ between MYPT2 WT and KO mice (Fig. 3*E*). However, transcript levels of *Myh7* and *Tnnt1* were significantly decreased in the knockout (Fig. 3*E*). These results demonstrate that, in a MLCK3 null background, MYPT2 KO mice exhibited lighter hearts and downregulation of genes involved in cardiac remodeling.

### MYPT2 knockout leads to improved cardiac performance in C57BL/6N mice

**Figure 4:**
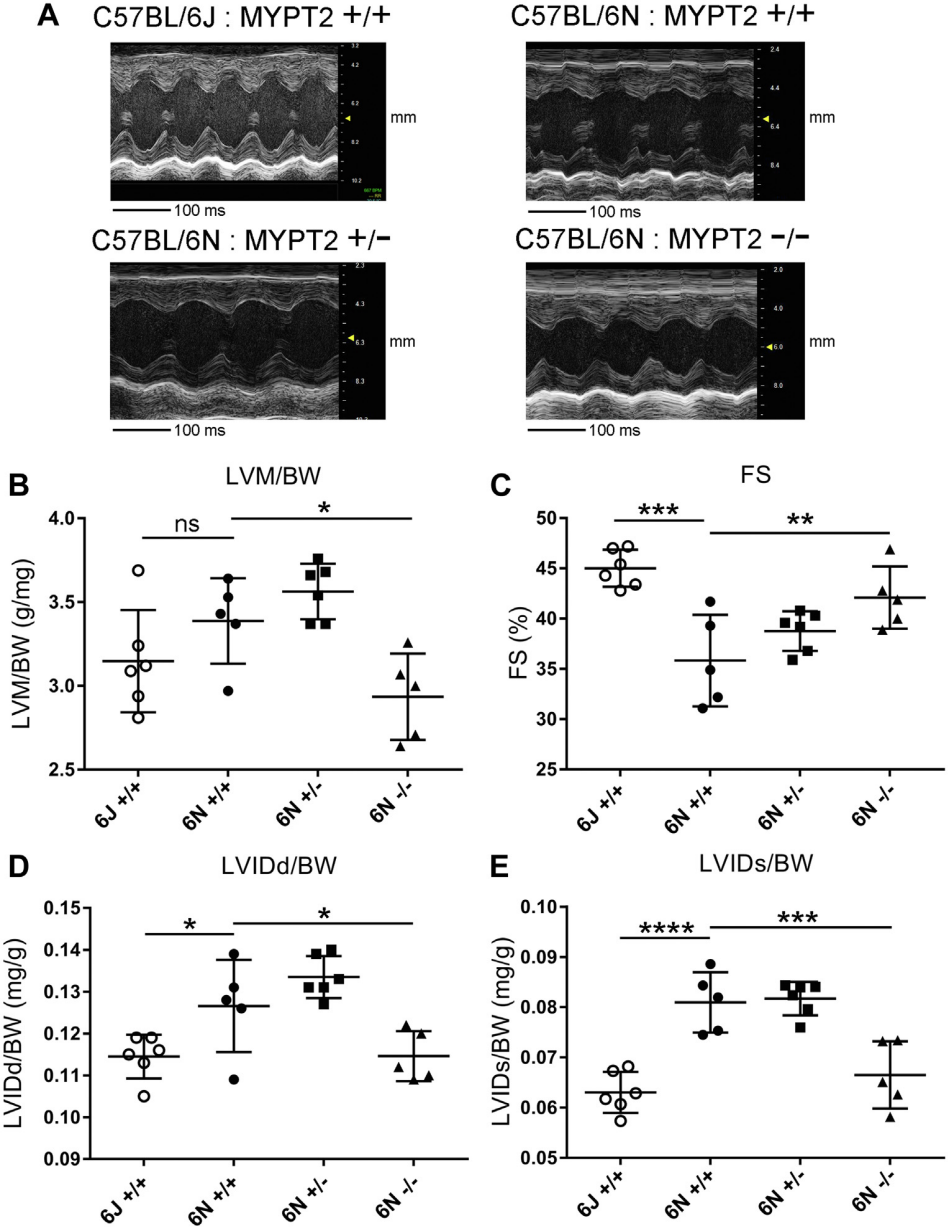
**MYPT2 knock-out leads to improved cardiac performance in C57Bl/6N mice.** Twenty-four-week-old male mice analyzed by cardiac echo. *A*, representative M-mode images. *B*, left ventricular mass (LVM) over body weight (BW). *C*, fractional shortening (FS). *D*, left ventricular inner diameter at diastole (LVIDd) over BW. *E*, left ventricular inner diameter at systole (LVIDs) over BW. Graphs represent means ± SD; ∗*p* > 0.05%, ∗∗*p* > 0.01, ∗∗∗*p* > 0.001, ∗∗∗∗*p* > 0.0001 using multiple comparisons; ns, nonsignificant. MYPT2, Myosin Phosphatase Targeting Subunit 2.

To assess the effect of MYPT2 knockout in the heart, we performed echocardiography on 24-week-old male MYPT2 WT, heterozygous, and KO mice in the C57BL/6N (MLCK3-deficient) background (Fig. 4). In parallel, we also examined WT C57BL/6J mice. C57BL/6J mice express MLCK3 and show no difference in the phosphorylation level of the main regulatory threonine residue in MYPT1 or MYPT2 when compared to C57BL/6N mice (Fig. S4). There was no difference in the heart rate or BW between the different groups (Fig. S5, *A* and *B*). We found a trend toward increased left ventricular mass (LVM)/BW in the C57BL/6N mice when compared to C57BL/6J (Fig. 4*B*). However, there was a significant decrease in LVM/BW in the MYPT2 KO mice compared to parental C57BL/6N mice (Fig. 4*B*). Compared to C57BL/6J mice, FS was decreased in the parental C57BL/6N mice (FS = 45% and 36%, respectively) and significantly increased when MYPT2 was absent in the C57BL/6N background (FS = 42%) (Fig. 4*C*). Left ventricular inner diameter at diastole and left ventricular inner diameter systole normalized to BW were highest in parental C57BL/6N mice, both compared to C57BL/6J mice (+MLCK3/+MYPT2) and MYPT2 KO C57BL/6N mice (-MLCK3/-MYPT2) (Fig. 4, *D* and *E*). There were no differences in the interventricular septum thickness in diastole or left ventricular posterior wall thickness in diastole (Fig. S5, *C* and *D*). Taken together, our results demonstrate that mild cardiac dysfunction observed in the absence of MLCK3 can be largely restored by deletion of MYPT2. They also highlight that MYPT2 plays a key role in regulating cardiac function *in vivo*.

## Discussion

**Figure 5:**
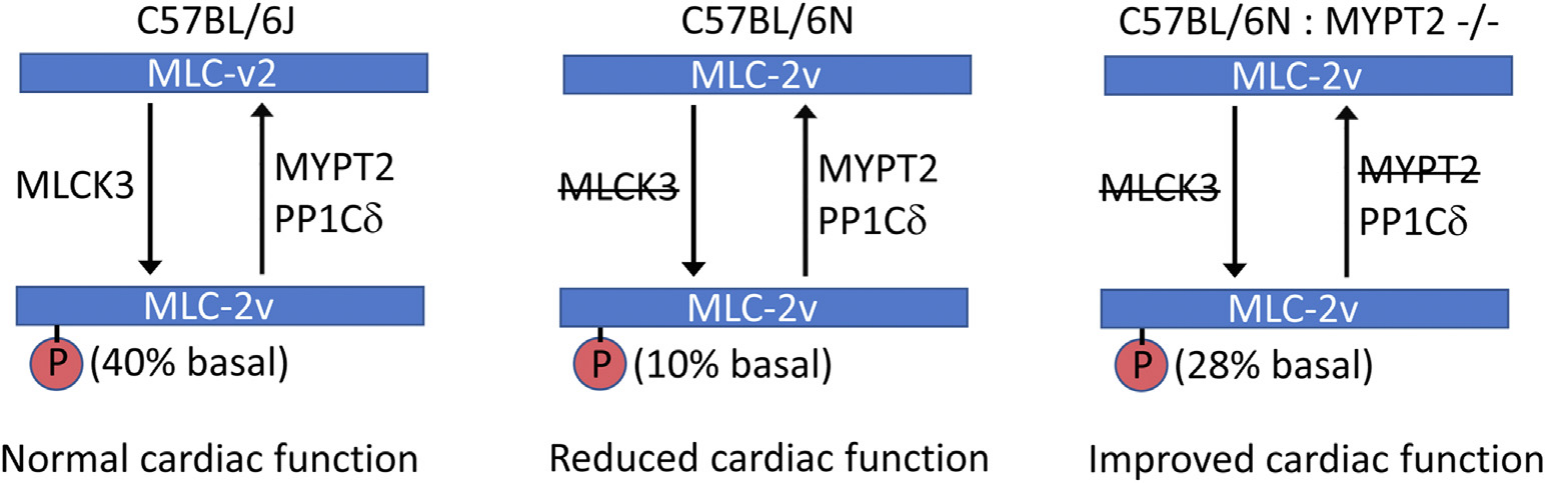
**Schema showing the proposed model for MYPT2 function.** In C57BL/6J mice with MLCK3 and MYPT2, the balanced activity of MLCK3-induced phosphorylation and MYPT2-mediated dephosphorylation leads to a basal MLC-2v phosphorylation of 40% and normal cardiac function. The lack of MLCK3 in C57BL/6N mice disrupts this balance, and the basal level of MLC-2v phosphorylation drops to ∼10%, causing reduced cardiac function. Knocking out MYPT2 in C57BL/6N mice increases MLC-2v phosphorylation to ∼28% and improves cardiac function. P, Ser^15^ phosphorylation. MYPT2, Myosin Phosphatase Targeting Subunit 2; MLCK, myosin light chain kinase.

The results from our current study can be summarized as shown in Figure 5. In mice containing MLCK3 and MYPT2 (*i.e.*, C57BL/6J with both kinase and phosphatase present), “basal” MLC-2v phosphorylation of 40% and “normal” cardiac function is observed. In C57BL/6N mice, which lack MLCK3 (the kinase), MLC-2v phosphorylation drops to 10% and cardiac function is compromised. However, knocking out MYPT2 (the phosphatase targeting subunit) in C57BL/6N mice leads to increased MLC-2v phosphorylation and improved cardiac function.

### MLCK3 KO studies

The finding that C57BL/6N mice lack MLCK3 provides an explanation for this strain’s predisposition to heart failure (20, 21). We also observed reduced cardiac function in C57BL/6N mice when compared to C57BL/6J mice, but function was improved by MYPT2 knockout, correlating with a restoration of MLC phosphorylation. In 2010, Ding *et al.* (9). created a hypomorphic MLCK3 allele (*Mylk3 gene*)—due to the presence of a neomycin cassette which was not removed after creation of a LoxP-targeted allele. In mice homozygous for this hypomorphic allele (MLCK3^neo/neo^), MLCK3 expression was undetectable by Western blot and the fraction of phosphorylated MLC-2v was reduced to ∼10% (compared to 40% in the corresponding WT mice which were in a mixed 129SvEv and C57BL/6 background). This level of MLC-2v phosphorylation is similar to what we see in the parental C57BL/6N mice. At 18 to 22 weeks, Ding *et al.* (9) found increased LVM and compromised cardiac function in mice homozygous for the hylomorphic allele *versus* WT mice (MLCK3^+/+^). In another study, Warren *et al.* (13) described mice with a germline MLCK3 deletion: under basal conditions at 12 weeks of age, the hearts were moderately enlarged with increased weight and also showed reduced FS and increased left ventricular volumes at diastole and systole. When these mice were subjected to transaortic constriction, they were highly susceptible to heart failure with increased dilation of the ventricles (at both systole and diastole) and reduced contractility compared to WT mice. In contrast, mice with cardiac myocyte-specific MLCK3 overexpression were protected from pressure-overload–induced cardiac dysfunction (13).

### MYPT family members and PP1Cδ targeting

There are three PP1C family members: PP1Cα, PP1Cγ, and PP1Cδ. MYPT1 and MYPT2 specifically interact with PP1Cδ, target it to myosin, and regulate its activity (15). We found that MYPT2 knockout leads to decreased levels of PP1Cδ in the heart. This is consistent with the finding that overexpression of MYPT2 in cardiac myocytes leads to increased PP1Cδ levels (19) and that knocking out MYPT1 in smooth muscles cells leads to decreased PP1Cδ levels (27). Thus, the presence of MYPT family members appears to stabilize PP1Cδ in cells. On the other hand, cardiomyocyte-specific PP1Cδ deletion does not lead to decreased levels of MYPT2 (28). We did not observe a change in PP1Cγ expression, and this might be expected as the latter does not interact with MYPT2 (15).

We found that murine hearts express about twice as much MYPT2 as MYPT1. Similar results have been reported by others (28). While MYPT2 knockout compensated for the absence of MLCK3, MLC-2v phosphorylation and cardiac dysfunction were not completely reversed. MLC-2v phosphorylation only reached ∼28%. It is possible that the remaining MLC-2v kinase (MLCK4 or ZIPK) has a more restricted ability to phosphorylate MLC-2v or that MLC-2v was still being actively dephosphorylated. The remaining MLC-2v dephosphorylation may be mediated by MYPT1-targeted PP1Cδ or by free PP1Cδ (or one of the other PP1C family members). The relative contribution of these kinases and phosphatases in regulating MLC-2v phosphorylation remains to be determined and would require additional mouse models. However, it is important to keep in mind that we determined only the average level of MLC-2v phosphorylation in whole heart extracts, but there are nonuniform MLC-2v phosphorylation patterns seen in histological and other studies (5, 13). Thus, in WT C57BL/6J mice, levels of MLC-2v phosphorylation are likely higher than 40% maximum at the apex and lower near the mid-cardiac region.

Severieva *et al.* (23) reported that MLCK3 phosphorylates human and rodent cardiac troponin I. Since troponin is a target of MLCK3, we checked if troponin I phosphorylation was altered in the MYPT2 KO mice, but we found that MYPT2 deletion had no effect. We also examined MYBPC3 phosphorylation in heart lysates by Phos-tag gel electrophoresis, for lack of available phosphospecific antibodies (25); we found no difference between WT and MYPT2 KO mice. We observed two bands on Phos-tag gels, representing differentially phosphorylated states of MYBPC3, in heart lysates obtained by extracting snap-frozen, pulverized tissue in urea buffer containing a phosphatase inhibitor cocktail. In contrast, Copeland *et al.* (29) observed 4 to 5 bands on Phos-tag gel electrophoresis, using MYBPC3 partially purified from heart extracts in phosphate buffer with NaF as a phosphatase inhibitor. It is possible that they observed more bands due to partial MYBPC3 dephosphorylation, which occurred during the purification process. It is also possible that the monoclonal antibody we used for our blots did not recognize all phosphorylated forms of MYBPC3.

### MLCK3, MYPT2, and cardiac gene expression

We found that expression of *Myh7* and *Tnnt1* were decreased in 12-week-old C57BL/6N MYPT2 KO mice when compared to parental controls. In humans and larger mammals, the predominant ventricular heavy chain is expressed from the *Myh7* gene, while in adult mice and rats, the predominant form is expressed from the *Myh6* gene (30). Nevertheless, *Myh7* expression levels increase in mouse models of heart dysfunction (9, 20). The decreased *Myh7* expression seen in MYPT2 KO mice is consistent with the observed smaller heart size and improved cardiac function. *Tnnt1* codes for slow skeletal troponin T, and its expression has been shown to increase in end-stage human heart failure (31). Williams *et al.* (20) found that *Myh7* and *Tnnt1* expression are higher in C57BL/6N mice than in C57BL/6J mice, and our findings are consistent with the MYPT2 knockout at least partially reversing the cardiac remodeling that occurs in the absence of MLCK3. Like Williams *et al.* (20), we saw no change in *Nppa* and *Nppb* expression under basal conditions. These genes code for atrial natriuretic peptide and brain natriuretic peptide, respectively, and their expression is known to increase in response to pressure overload (32). In contrast to the findings of Williams *et al.* (20), Ding *et al.* (9) found increased *Nppb* expression with no change in *Myh7* expression in 18 to 22-week-old MLCK3^neo/neo^ mice. In addition, while we saw reduced *Myh7* expression, and no change in *Nppb* expression in MYPT2 KO mice (when compared to WT parental C57BL/6N mice), in cardiac myocytes overexpressing MYPT2, *Myh7* and *Nppb* gene expression both increased compared to nontransgenic controls (19). The differences between our results and the results of other groups may be due to subtle genetic differences in the mouse strains and/or differences in the age of the mice used in the studies.

## Conclusion

In summary, this is the first report describing the cardiac phenotype of global MYPT2 KO mice. Our study concludes that knocking out MYPT2 in an MLCK3-deficient background significantly increased MLC-2v phosphorylation, leading to improved cardiac function. Importantly, while both MYPT1 and MYPT2 are expressed in heart tissue, MYPT1 cannot compensate for the loss of MYPT2 in controlling MLC-2v phosphorylation. Overall, our findings highlight the important role MYPT2 plays in regulating cardiac performance *in vivo*.

## Experimental procedures

### Mice

MYPT2 KO mice were generated by the Mutant Mouse Resource Center and obtained from the Jackson laboratory (MMRRC Stock No: 46179-JAX). C57BL/6NJ mice (Jax Strain #:005304) and C57BL/6J mice (Jax strain #:000664) were obtained from the Jackson Laboratory (to avoid confusion, throughout the paper, we refer to the C57BL/6NJ mice as C57BL/6N). Mice used (C57BL/6NJ-*Ppp1r12b*^*em1(IMPC)J*^/Mmjax) to generate data were either 12 or 20 to 28 weeks of age. They were housed in a temperature-controlled environment with a 12/12 h light/dark cycle. Mice were weaned at day 18 and genotyped by PCR and agarose gel electrophoresis using genomic DNA from approximately 2 mm of clipped mouse tail. The genomic DNA was obtained by incubation of the tail in 75 μl of 25 mM NaOH/0.2 mM EDTA at 98 °C for 1h. The tube was then cooled to room temperature and 75 μl of 40 mM tris HCl (pH 5.5) was added. PCR reactions were run using HotStart Taq polymerase (BioPioneer, catalog #: MAT-4) and the following primers: 5′-TGTTTTCCCTACTTGCTGTG-3′ and 5′-GCTTGTGTTCTGCTGTTTG-3′. The WT allele produced a 775 bp product and the mutant allele produced a 195 bp product. To check for the mutation which prevents MLCK3 production, we amplified genomic DNA using PCR and the following primers: ATGstartF 5′-ACAGCTTACACGGCTCTTGC-3′ and ATGstartR 5′- ACCCAACACACTGCTGA-AAAC-3′. The PCR produced was gel purified using QIAquick Gel Extraction Kit (Qiagen) and sequenced using ATGstartF as a primer. All animal experiments were approved by the Institutional Care and Use Committee of the University of California, San Diego.

### Materials

Dulbecco’s Modified Eagle Medium was from Cellgro. Fetal bovine serum was from Sigma-Aldrich. Restriction enzymes were from New England Biolabs. Protease inhibitor cocktail was from EMD Millipore Corporation (Cat#: 539131) and phosphatase inhibitor cocktail was from Cell Signaling Technology (Cat#: 5870). Horseradish peroxidase (HRP)-conjugated anti-Flag antibody was from Sigma-Aldrich (Cat#: A8592). Rabbit anti-MYPT1/2 was from Abcam (Cat#: ab32519). Anti-PPP1CB and anti-Phospho-MLC-2v (Ser15) antibodies were from Thermo Fisher Scientific (Cat#: PA5-28225 and PA5-104265, respectively). Anti-PPP1CC antibody was from Origene (Cat#: TA308937). Anti-Phospho-MYPT1 (Thr696) antibody was from Cell Signaling Technology (Cat#: 5163). Anti-MYBPC3 (Cat#: sc-137237) and HRP-conjugated anti-β-actin antibody (Cat#: sc-47778-HRP) were from Santa Cruz Biotechnology. Peroxidase-conjugated Goat anti-Rabbit IgG was from Jackson ImmunoResearch (Cat#: 111-035-003). SuperSignal West Pico Plus was from Pierce (Cat#: 34580). IRDye 680RD Goat anti-mouse and IR DYE 800CW Goat anti-Rabbit and Intercept Blocking Buffer were from LI-COR. Other general chemicals and supplies were from Thermo Fisher Scientific or Sigma-Aldrich.

### Cell culture

293T cells were grown in Dulbecco's Modified Eagle Medium (Cellgro) with 10% fetal calf serum (Sigma-Aldrich) at 37 °C with 5% CO_2_ in a water jacketed incubator. Stock cells were grown in 10 cm tissue culture dishes, and cells were split every two to 3 days using 0.25% trypsin/EDTA (Gibco).

### Expression vectors and transfection

Mouse MLC-2v was cloned by PCR using reverse transcribed total RNA isolated from mouse heart tissue as a template. The coding sequence was inserted pQTEV in frame with the N-terminal His-tag. DNA clones for MYPT1 (BC111752), MYPT2 (BC144699), MBS85 (BC010628), PPP1CB (BC002697), PPP1CC (BC014073) were obtained from Horizon Discovery. The MBS85 clone was not full-length and PCR was used to produce an N-terminal sequence optimized for expression in mammalian cells. The expression vectors of MYPT1, MYPT2, and MBS85 were engineered with N-terminal Flag-tags. The expression vectors for PPP1CB and PPP1CC were engineered to have N-terminal Myc-tags. The D565A mutation in MLCK3 was produced using overlapping extension PCR (33, 34). Expression constructs were then transfected into 293T cells using Lipofectamine^2000^ (Thermo Fisher Scientific). For each transfected sample, DNA/Opti-MEM and Lipofectamine^2000^/Opti-MEM mixtures were incubated for 5-min at room temperature. The two incubations were mixed and incubated for an additional 20 min at room temperature. The mixtures were then added to 293T cells and incubated for 20 to 24 h before harvesting.

### Western blotting

For transfected 293T cells, lysates were prepared by aspirating the media from the cells and directly scraping in ice cold Lysis Buffer 1 (PBS, 0.1% NP40, and protease inhibitor cocktail). Lysates were cleared by centrifugation at 16, 000*g* for 10 min at 4 °C, and cleared lysates were mixed 2:1 in 3× in SDS sample buffer. Equal amounts of lysates were separated by SDS-PAGE.

For transgenic MYPT2 KO mice, frozen tissues were pulverized on dry ice, resuspended in RIPA buffer (EMD Millipore, Cat# 20-188) with protease (Calbiochem, Cat#: 539131) and phosphatase (Cell Signaling, Cat#: 5870) inhibitors. The suspension was sonicated twice for 15 s on ice. Lysates were cleared by centrifugation at 16, 000*g* for 10 min at 4 °C. Proteins were quantified using a Bradford assay, and 6 or 60 μg protein from each sample was separated by SDS-PAGE.

Proteins were transferred to polyvinylidene difluoride membrane and blocked in 5% milk in Tris-buffered saline-01% Tween 20. For blots using IR dye secondary antibodies, proteins were transferred to Immobilon-FL polyvinylidene difluoride membrane and blocked in Intercept Blocking Buffer (LI-COR).

Western blots were performed with antibodies directed against Flag-epitope (1:5000), MYPT1/2/MBS85 (1:1000), PP1Cδ (1:1000), PP1Cγ (1:1000), and HRP-conjugated β-actin (1:10000). HRP-conjugated secondary antibodies were used at 1:5000, and IR-dye secondary antibodies were used at (1:10000). Antibodies were diluted in the blocking buffer used for each condition. HRP blots were visualized using SuperSignal West Pico Plus chemiluminescent Western blotting substrate, and IR blots were visualized using an Odyssey Imaging System (LI-COR).

### Quantification of MLC-2v phosphorylation

MLC phosphorylation was determined using urea/glycerol PAGE, using a protocol derived from (35). Briefly, 20 mg pulverized heart tissue was resuspended in 240 μl resuspension buffer, which consisted of 1.3 ml glycerol buffer (60% glycerol and 10× protease/phosphatase inhibitor cocktail) and 5 ml urea buffer (2.71 g Urea, 50 mM Tris (pH 8.6), 300 mM glycine, 5 mM DTT). Resuspended tissue was incubated at 60 °C for 4 min and Dounce homogenized. Lysates were cleared by centrifugation at 16, 000*g* for 10 min at 4 °C. Protein concentration was determined by Bradford assays. Samples were diluted to 300 μg/ml in resuspension buffer containing 0.004% bromophenol blue. The stacking gel consisted of 20% glycerol, 5% acrylamide, 0.25% bis-acrylamide, 50 mM glycine, and 25 mM Tris (pH 8.6). The resolving gel consisted of 40% glycerol, 10% acrylamide, 0.5% bis-acrylamide, 50 mM glycine, and 25 mM Tris (pH 8.6). The gel was pre-run for 1 h in 23 mM glycine, 20 mM Tris (pH 8.6). After the pre-run, 5 mM DTT was added to the upper chamber, and 6 μg protein/lane was loaded onto the gel. Gel was run for 1 h at 400 V. Proteins were transferred to nitrocellulose membrane 1.5 h at 300 mA in 10 mM Na_2_HPO_4_ (pH 7.6) and then fixed with 15% glutaraldehyde in PBS for 15 min at room temp. Membranes were blocked in 5% milk/Tris-buffered saline-01% Tween 20.

### *In vitro* phosphorylation of MLC-2v

His-tagged MLC-2v was produced in DH5α *Escherichia coli* and purified using metal affinity chromatography. Flag-tagged WT ‘active’ and D565A ‘dead’ Δ491 MLCK3 were produced in transiently transfected 293T cells and purified using anti-Flag affinity gel followed by elution with Flag peptide. Nonradioactive reactions contained 500 ng MLC-2v, 20 ng d Δ491 MLCK3, 25 mM Hepes (pH 7.4), 50 mM NaCl, 2 mM MgCl_2_, 2 mM DTT, 1 mM Ca^2+^, and 500 μM ATP with or without 1 μg calmodulin (Sigma: Cat#: 2008694). Reactions were run for 10 min at 30 °C and stopped by adding an equal volume of 2× SDS sample buffer containing 50 mM EDTA. Radioactive reactions were run under the same conditions, except cold ATP was reduced to 100 μM and 40 μCi ^32^P-γ-ATP was added. Reactions were separated by SDS-PAGE, transferred to Immobilon, and subjected to autoradiography or immunoblotting with the indicated antibodies.

### Phospho-tag gel electrophoresis

To measure MYBPC3 phosphorylation levels, pulverized heart tissue was resuspended in 8 M Urea, 2% CHAPS, 20 mM DTT, protease/phosphatase inhibitor cocktail and 0.004% bromophenol blue. The mixture was sonicated twice for 30 s at 4 W, and lysates were cleared by centrifugation at 16, 000g for 10 min. Protein concentration was determined by Bradford assays, and 10 μg of protein per sample was separated on a 5% Phos-tag gel (FUJIFILM Wako Chemicals). The gel contained 30 μM Phos-tag solution and 60 μM MnCl_2_. After running, the gels were soaked for 10 min in transfer buffer containing 1 mM EDTA and 10 min in transfer buffer alone. Proteins were transferred to Immobilon membranes and analyzed by immunoblotting. Bands were quantified using ImageJ. Relative MYBPC3 phosphorylation was expressed as the ratio of the signal intensity in the upper band over the total signal in both bands.

### Quantitative RT-PCR

Hearts were snap-frozen in liquid nitrogen within 5 min of euthanasia. Frozen hearts were pulverized and resuspended in Trizol (Molecular Research Center, TR118), and total RNA was isolated as per the manufacturer’s instructions. Total RNA was reverse-transcribed using iScript cDNA synthesis kit (Bio-Rad). Quantitative PCR was performed using a MX3005P real-time PCR detection system with Brilliant II SYBR Green Mix (Agilent Technologies) as described (36). The primers used are listed in Table S1. All primers were intron-spanning and were tested with serial complementary DNA dilutions. Relative changes in mRNA expression were analyzed using the comparative 2^−ΔΔCt^ method.

### Echocardiography

To perform echocardiography, 24-week-old male mice were anesthetized with 0.5% isoflurane. Electrocardiograms were monitored with probes inserted into the upper and lower torso. Cardiac function was recorded with a VisualSonics, SonoSite FUJIFILM, Vevo 2100 ultrasound system with a linear transducer 32–55 MHz by a single, highly experienced operator who was blinded to the genotype of the mice.

### Statistical analysis

All statistical analysis was performed using Graphpad Prism. Data are reported as mean ± SD. Comparisons between multiple groups were done using one-way ANOVA with Sidak's multiple comparisons test. Comparisons between two groups were performed by two-tailed Student’s *t* test. A value of *p* < 0.05 was considered statistically significant.

## Data availability

All supporting data is in the manuscript.

## Supporting information

This article contains supporting information.

## Conflict of interest

The authors declare that they have no conflicts of interest with the contents of this article.
