# Supplementary material for: Phosphatase regulatory subunit MYPT2 knockout partially compensates for the cardiac dysfunction in mice caused by lack of myosin light chain kinase 3
Source: J Biol Chem. 2023 Mar 7;299(4):104584. doi: 10.1016/j.jbc.2023.104584 (PMC10124902; doi:10.1016/j.jbc.2023.104584)

Supporting Information

Phosphatase regulatory subunit MYPT2 knockout partially compensates for the cardiac dysfunction in mice caused by lack of myosin light chain kinase 3

Tingfei Hu, Hema Kalyanaraman, Renate B. Pilz and Darren E. Casteel

This file contains Table I (listing primers used for qPCR) and supplemental data which complements the data shown in the main manuscript.

Supplemental Figure Legends

Supplemental Figure 1. MYPT antibody specificity test. *A*, Cell lysates from 293T cells expressing Flag-tagged MYPT1, MYPT2, or MBS85 were separated by SDS-PAGE and probed with mouse anti-Flag antibody followed by an IR Dye 680RD anti-rabbit antibody. Protein expression levels were quantified using a Li-COR Odyssey imaging system. *B* The same blot as shown in A was probed with rabbit anti-MYPT1/2 antibody followed by an IR Dye 800CW anti-rabbit antibody. Proteins were quantified as described for A.

Supplemental Figure 2. MYPT1/2 expression in brain and skeletal muscle. Western blots of lysates from 12-week-old wild-type (+/+), heterozygous (+/-) and homozygous (-/-) MYPT2 knock-out mice using pan-MYPT and β-actin antibodies. *A*, Brain. *B*, Skeletal muscle (Sk.M.).

Supplemental Figure 3. MLCK3 start codon sequencing and MLC-2v sequence alignment. *A*, Mylk3 sequence alignment around the MLCK3 start codon. The endogenous ATG start codon is shown in bold-italic. The point mutation in C57BL/6N mice is colored blue, and the new out of frame start codon is underlined. The lower panel shows a sequencing trace from the parental C57BL/6N mice used in our study. *B*, Mouse and human MLC-2v are 166 amino acids long. Sequence alignment of the first 30 amino acids shows four amino acid differences (colored blue) just N-terminal to the Ser^{15} (underlined).

Supplemental Figure 4. MYPT1 and MYPT2 phosphorylation at the regulatory Thr residue is similar in C57BL/6J and C57BL/6N mice. ***A*,** Heart lysates from 12-week-old mice were resolved by SDS-PAGE, transferred to Immobilon and probed with antibodies that detect pThr696-MYPT1/pThr646 MYPT2 or total MYPT1/2, as indicated (ns = a non-specific band that migrates above MYPT1). *B*, Quantification of data from three mice as described in panel A.

Supplemental Figure 5. Cardiac echo data from C57BL/6N MYPT2 knock-out mice. Twenty-four-week old male mice analyzed by cardiac echo. *A*, Heart rate. *B*, Body weight. *C*, Interventricular septal thickness at end diastole (IVSd). *D*, Left ventricular posterior wall thickness in diastole (LVPWd).

Supplemental Table 1: Primers Used for Quantitative RT-PCR

All primer pairs were tested with serially-diluted cDNAs and were intron-spanning

Target	Sense (5’ to 3’)	Anti-sense (5’-3’)
Nppa	TCTTCCTCGTCTTGGCCTTT	GCTTCCTCAGTCTGCTCACT
Nppb	AGCCAGTCTCCAGAGCAATT	CGGTCTATCTTGTGCCCAAAG
Myh7	GTGAAGGCCTACAAGCGC	ACCAATGTCCCGGCTCTTG
Tnnt1	GCCCTTGAACATCGACTACA	TCAACTTCTCCATCAGGTCAAA
Hprt	CCAGACAAGTTTGTTGTTGGAT	GCTTTGTATTTGGCTTTTCCA

Supplemental Figure 1

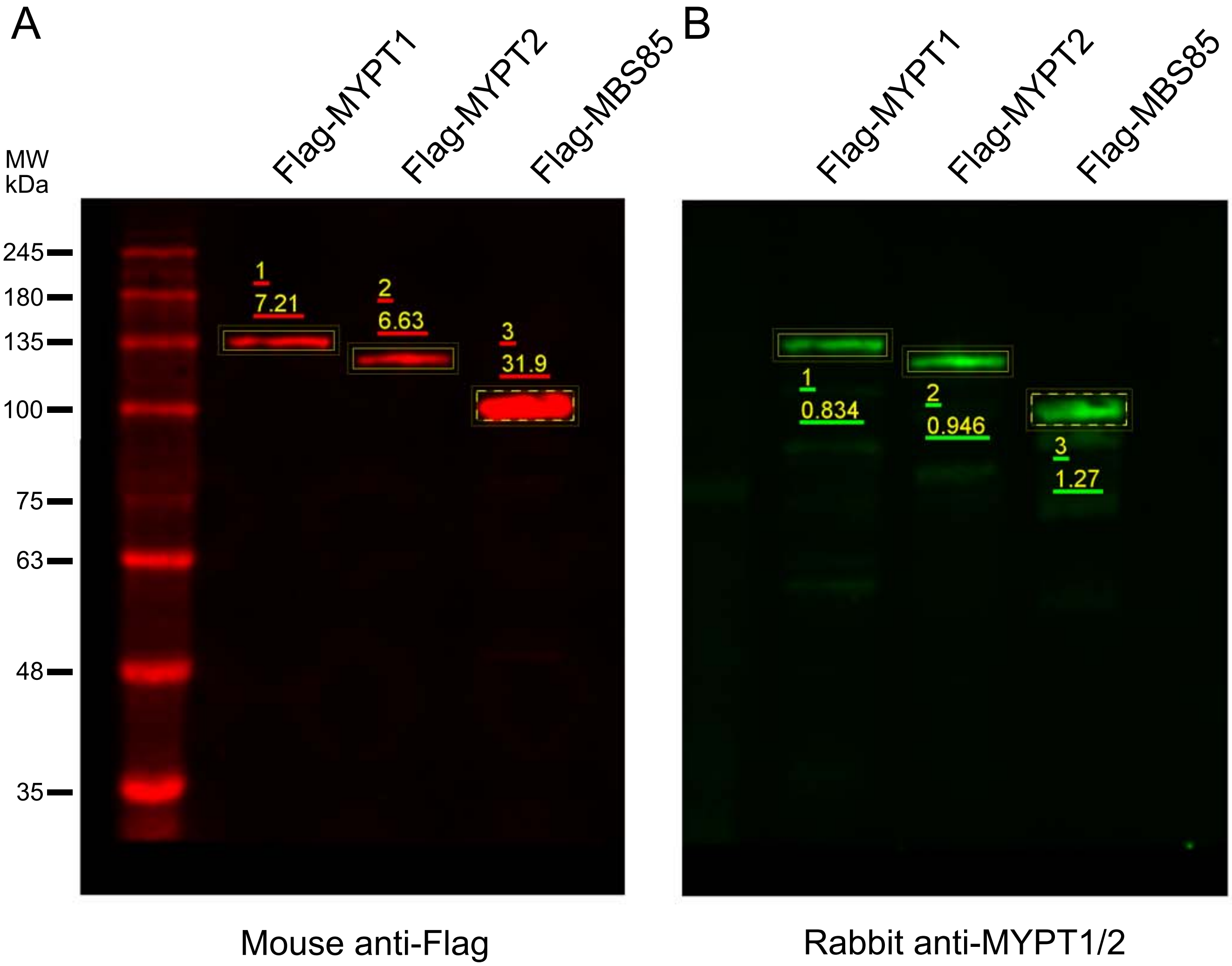

Supplemental Figure 2

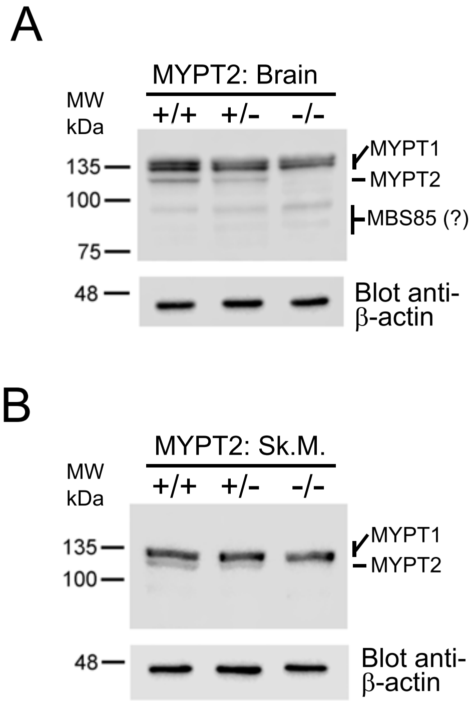

Supplemental Figure 3

A

6J: CCCGAATTGGG***ATG***TCAGGA
6N: CCCGAA<u>ATG</u>GG***ATG***TCAGGA

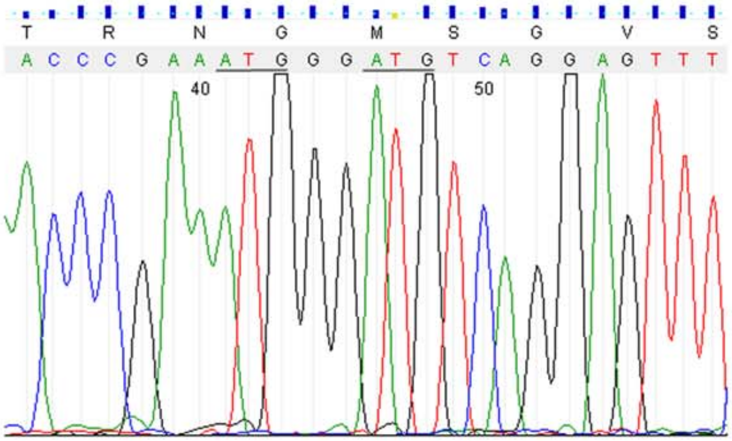

B

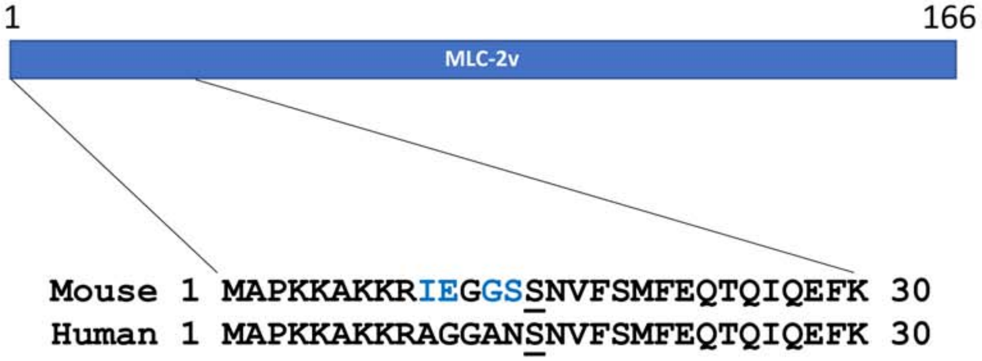

Supplemental Figure 4

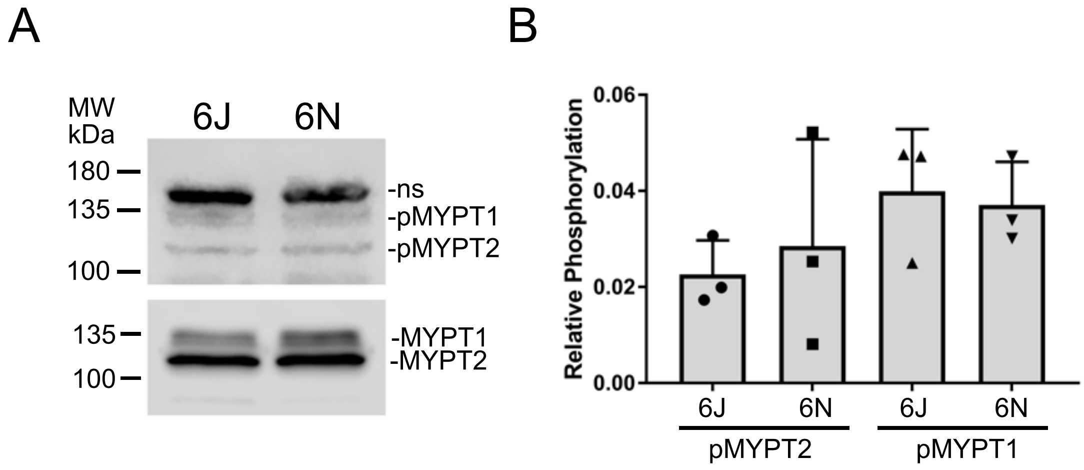

Supplemental Figure 5

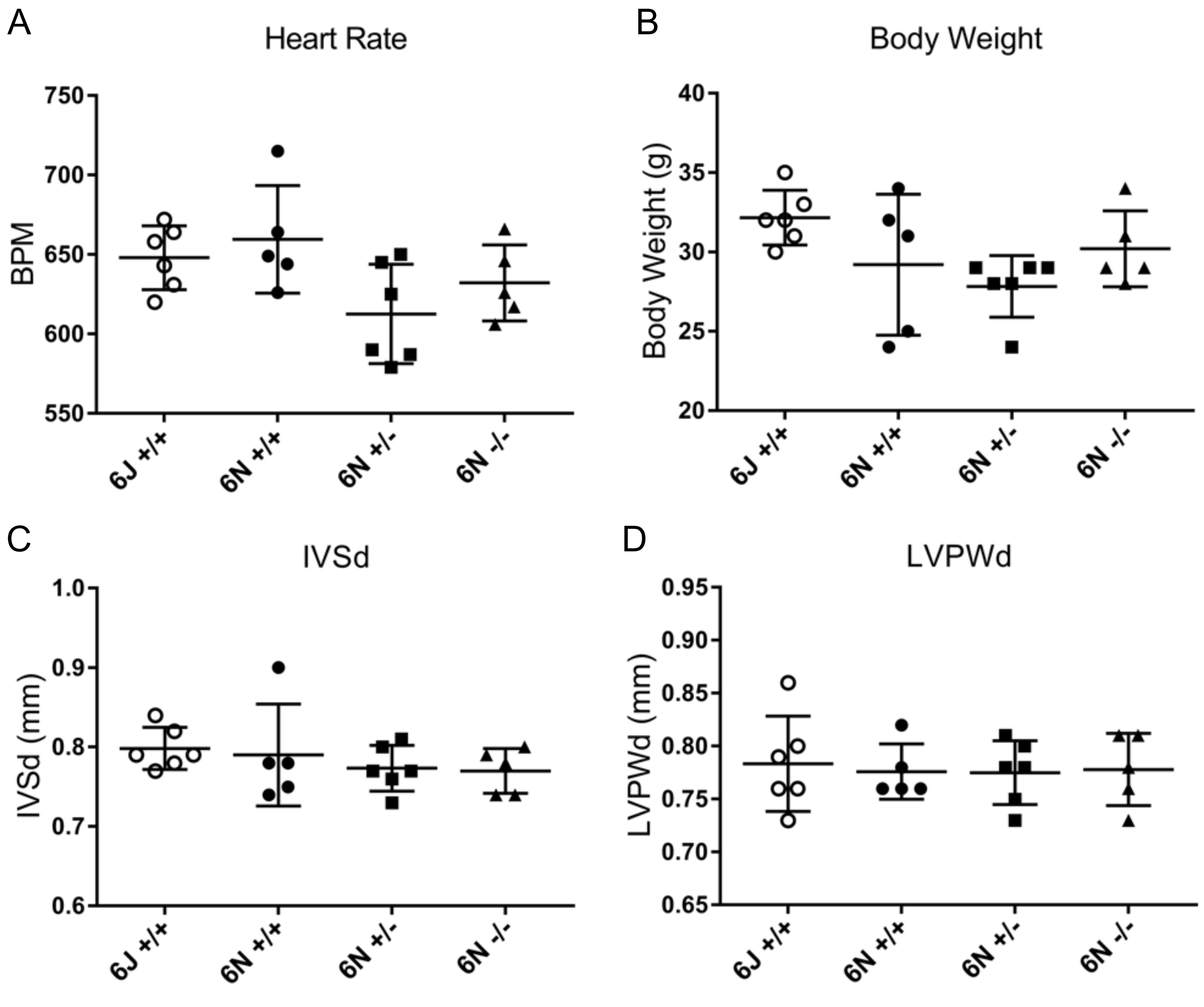

Supplement: Supplemental Figures S1–S5 and Table S1 [file mmc1.pdf]
